# Evaluating urinary metabolic profiles with wildland-urban-interface (wui) fire exposure among male firefighters: a comparison with municipal structure fires (msf)

**DOI:** 10.1186/s12940-025-01239-7

**Published:** 2025-11-17

**Authors:** Tuo Liu, Melissa A. Furlong, Justin M. Snider, Shawn Beitel, Catherine E. Mullins, Douglas I. Walker, Jaclyn M. Goodrich, Derek J. Urwin, Jamie Gabriel, Jeff Hughes, John J. Gulotta, Miriam M. Calkins, Yiwen Liu, Frank A. von Hippel, Paloma Beamer, Jefferey L. Burgess

**Affiliations:** 1 Department of Community, Environment, and Policy, Mel and Enid Zuckerman College of Public Health, University of Arizona, 295 N Martin Ave, Tucson, US; 2 Department of Epidemiology and Biostatistics, Mel and Enid Zuckerman College of Public Health, University of Arizona, Tucson, US; 3 School of Nutritional Sciences and Wellness, University of Arizona, Tucson, US; 4 Gangarosa Department of Environmental Health, Rollins School of Public Health, Emory University, Atlanta, GA USA; 5 Department of Environmental Health Sciences, School of Public Health, University of Michigan, Ann Arbor, MI USA; 6 Chemistry and Biochemistry Department, University of California at Los Angeles, Los Angeles, CA USA; 7 Los Angeles County Fire Department, Los Angeles, CA USA; 8 Orange County Professional Firefighters Association, Tustin, CA USA; 9 Tucson Fire Department, Tucson, AZ USA; 10 National Institute for Occupational Safety and Health, Centers for Disease Control and Prevention, Cincinnati, OH USA

**Keywords:** WUI fires, Firefighters, Urinary metabolomics, Health risk

## Abstract

**Background:**

Firefighters have frequent exposure to carcinogens and an increased risk of cancer. Wildland-urban interface (WUI) fires, which involve both structures and undeveloped wildland fuels, pose unique challenges to the health of firefighters. However, the extent of health risks associated with these fires remains underexplored.

**Objectives:**

This study aims to identify altered urine metabolites and metabolic processes among male firefighters that were associated with WUI fires as compared with municipal structure fires (MSF).

**Methods:**

Untargeted metabolomic profiling was applied to pre-exposure (baseline) and postfire urine samples collected from firefighters responding to WUI and MSF fires. Differential analysis was conducted by fitting linear mixed effects regression models on preprocessed ion intensity and exposure status while adjusting for demographic covariates. Differential metabolites by post-exposure status were identified using a false discovery rate (FDR) threshold of < 0.05. Pathway analysis was performed to identify pathways that were significantly perturbed at a Bonferroni adjusted p-value < 0.05 level. We conducted differential and pathway analyses in both the WUI and MSF cohorts and compared the two fire types in terms of the number of differentially expressed metabolites and patterns of metabolic pathway enrichment.

**Results:**

Eighty-five firefighters contributed paired baseline and post-fire samples from WUI events, and 98 firefighters contributed paired baseline and post-fire samples from MSF events. We performed metabolic profiling on baseline and postfire urine samples from WUI and MSF using four modes: HILIC(-), HILIC(+), C18(-), and C18(+) and identified metabolites against an in-house library. We identified 244, 297, 320, and 266 level-1 metabolites from the four respective modes. In the statistical analysis, the main model identified a total of 176 differential metabolites from WUI fires. For MSF, the model identified a total of 652 differential metabolites from the four respective modes. Most metabolites with significant changes after a WUI fire also changed significantly after an MSF event. Two metabolic pathways were significantly enriched after WUI fires, while 7 pathways were significantly enriched after MSF exposure and 2 pathways overlapped between the two types of fires.

**Conclusion:**

Fire exposure induces numerous metabolic perturbations in firefighters responding to WUI fires, potentially contributing to their elevated cancer risk. Although individual metabolites changed in a similar fashion across both WUI and MSF, MSF were associated with an increased number of metabolite changes and some of the enriched pathways differed between exposures to WUI fires vs. MSF. These findings suggest that WUI and MSF exposures may share common biological responses while also posing unique health risks to firefighters.

**Supplementary Information:**

The online version contains supplementary material available at 10.1186/s12940-025-01239-7.

## Introduction

The wildland-urban-interface (WUI) is a transitional zone where developed residential and commercial areas intermingle with undeveloped wildland and vegetation. According to the United States Fire Administration, urban areas have expanded into wildlands in the continental United States at an annual rate of 809,371 hectares over the past decade, affecting more than 60,000 communities and associated with destruction of an average of 3,000 structures annually by WUI fires [1]. Firefighters are routinely exposed to known and probable carcinogens, such as benzene [2], polycyclic aromatic hydrocarbons (PAHs) [2, 3], and per- and polyfluoroalkyl substances (PFAS) [4] among others during fire responses. The occupation of firefighting was recently classified as a Group 1 carcinogen by the International Agency for Research on Cancer (IARC) [5]. Similar to municipal structure fires (MSF), wildland fires burn biomass and produce toxic smoke that induces oxidative stress [6], impairs lung function [7], and causes inflammatory responses [8–10]. These effects are associated with long-term health manifestations such as cardiovascular diseases [11] and cancers [5, 12, 13]. In addition to airborne contaminants from wildland fires, WUI fires can produce a range of differing yet more toxic chemicals from the combustion of structures and vehicles including hydrogen cyanide (HCN), volatile organic compounds (VOCs), and toxic metals [14–16]. Furthermore, the structures that are burned in WUI fires produce similar chemicals to wildland fires but at different levels; for example, PAHs from burning structures are emitted at several orders of magnitude greater levels than occurs in wildfires [17].

Due to the mingling of urban environments and natural vegetation in undeveloped wildlands, and the associated complexity of occupational health concerns, WUI fires present challenges for local fire management agencies. Unlike the heavier, insulating personal protective equipment (PPE) used in structure firefighting, which includes a three-layered ensemble of protective textiles and self-contained breathing apparatuses against intense heat and toxic smoke, WUI/Wildland single layering is lighter and less insulating to accommodate a greater need for mobility and breathability in harsh outdoor conditions. Consequently, firefighters responding to WUI events may be exposed to higher doses of toxic chemicals due to the reduced level of protection. As WUI fires increase in frequency and intensity [18], more firefighters are being exposed, highlighting the need to understand associated health risks.

While previous research has characterized chemical exposures for firefighters [2, 3, 6, 14–17, 19] and presented epidemiological evidence of health effects associated with occupational firefighting [11, 20–23], exposures from WUI fires and their associated health effects have been understudied. It remains unclear how WUI fires affect the metabolic equilibrium of firefighters and how this could contribute to chronic health conditions. In particular, understanding how the metabolomic response to WUI fires differs from the response to MSF can help identify biological mechanisms that may contribute to differing risks.

Using untargeted, high-resolution metabolomics (HRM), this study aims to investigate the impact of WUI fires on the metabolic profiles of firefighters and to explore potential health risks, as well as to compare the biological effects of exposure to WUI fires with exposure to MSF alone. We hypothesize that a set of altered metabolites exist that are associated with adverse health outcomes for firefighters. Furthermore, we anticipate that WUI fires and MSF share common metabolic disruptions, while each induces distinct alterations in metabolic functions that pose unique health risks specific to each fire type.

## Methods

### Study population and sample collection

The study population included 87 firefighters enrolled in the Fire Fighter Cancer Cohort Study (FFCCS) from two Southern California county fire departments whose response duties include both MSF and WUI fires, using a baseline and post-exposure sampling design. This study also included 100 male firefighters responding to MSF from the Tucson Fire Department to provide a comparison with firefighters responding to MSF alone. The firefighters responding to WUI fires were recruited in collaboration with local fire departments following the informed consent and biological sampling procedures of the FFCCS, as previously described [24, 25]. The municipal structural firefighters were enrolled as part of a similar prior study specific to Tucson Fire Department, as previously described [3]. For WUI firefighters, all baseline urine samples were collected in September 2019 except for two samples collected in May 2019, and all post-fire samples were collected 3–5 h after a fire in October 2019. All MSF urine samples were collected 3–4 h after a fire between October 2015 and December 2018. Baseline and postfire urine samples from structure firefighters were analyzed in the same laboratory environment as were the WUI samples. Urine samples were transported on ice and stored at −80 °C at the University of Arizona until processing and analyses.

### Sample preparation

Urine samples were processed in batches that included both study samples and quality assurance/quality control (QA/QC) samples using an Opentron OT2 automated liquid handler and 96-well plates. Before analysis, the samples were thawed at 4 °C. A 30 µL aliquot of urine was then mixed with 90 µL of acetonitrile containing [13]C-labeled internal standards. The mixture was vortexed for 2 min, left to equilibrate at 4 °C for 30 min, and subsequently centrifuged at 3,220×g for 45 min at 4 °C. Following centrifugation, two 30 µL aliquots of the supernatant were transferred to 96-well plates, each containing either 60 µL of water (for Reverse Phase Chromatography (C18)) or 60 µL of a 1:1 acetonitrile/water solution (for Hydrophilic Interaction Liquid Chromatography (HILIC)). These were vortexed for another 2 min and stored in a refrigerated autosampler until they were analyzed.

### High-resolution metabolomics

All WUI urine samples were sent on ice to the Comprehensive Laboratory for Untargeted Exposome Science at Emory University for high-resolution metabolic profiling. Our previous study on MSF was profiled using a different HRM platform. To facilitate the WUI and MSF comparison, we re-analyzed urine samples collected from firefighters before and after MSF exposure using the same HRM platform as WUI fires and compared these data with new analyses of urine samples collected from firefighters before and after their deployment against WUI fires.

HRM was performed using two systems configured for either C18 or HILIC. These systems consisted of a Vanquish Duo Ultra Performance Liquid Chromatography (UPLC) unit (Thermo Fisher Scientific, Rockford, IL, USA) paired with an Exploris 120 High-Resolution Mass Spectrometry (HRMS) system (Thermo Fisher Scientific, Rockford, IL, USA). The LC column temperatures were maintained at 40 °C for HILIC and 30 °C for C18, while the autosampler was kept at 5 °C. Samples underwent analysis via dual column chromatography with mobile phases tailored for optimal positive or negative ionization. For C18 chromatography, a Higgins TARGA C18 5 μm 50 × 2.1 mm column (Higgins Analytical, Inc., Mountain View, CA, USA) was used in both positive and negative ionization modes. HILIC chromatography utilized a SeQuant ZIC-HILIC 3.5 μm 50 × 4.6 mm column (Merck KGaA, Darmstadt, Germany) for positive mode and an XBridge Amide 3.5 μm 3.0 × 50 mm column (Waters Corporation, Milford, MA, USA) for negative mode. The mobile phase (MP) for C18 analysis consisted of water with 0.1% formic acid (MP-A) and acetonitrile with 0.1% formic acid (MP-B) for positive mode, and 10mM ammonium acetate in water (MP-B) paired with a 97.5/2.5 (v/v) acetonitrile/water mixture (MP-A) for negative mode. For HILIC, the mobile phases included water with 0.1% formic acid (MP-B) and acetonitrile with 0.1% formic acid (MP-A) for positive mode, and 10mM ammonium acetate in water at pH 9.5 (MP-B) with a 97.5/2.5 (v/v) acetonitrile/water mixture (MP-A) for negative mode. Flow rates ranged from 0.3 mL/min to 0.6 mL/min, with a total run time of 7.5 min.

Resolutions for MS and ddMS2 were set at 120,000 FWHM (at m/z 200, at 3 Hz) and 30,000 FWHM (at m/z 200, at 12 Hz), respectively; internal calibration was performed scan-to-scan. The scan range was set from 85 to 850 m/z. The automatic gain control (AGC) target and maximum injection time in full-scan MS settings were set to Standard and Auto, respectively. This allowed the instrument to automatically adjust the injection time for an ion count of ~ 1 × 10^6^ for MS and 1e^5^ for ddMS2. The TopN (N, the number of topmost abundant ions for fragmentation) was set to 4, and collision energy (NCE) was set to 20, 40, and 60. A heated electrospray ion (ESI) source was used. The spray voltage was set at 3.5 kV for positive mode and 2.5 kV for negative mode. The capillary temperature and the auxiliary gas heater temperature were set at 325 and 350 °C, respectively. Sheath gas and auxiliary gas flow rate were set at 50 and 10 (in arbitrary units), respectively. The Funnel RF level was set to 70.

### Feature preprocessing and metabolite annotation

Following analysis of all study and QA/QC samples, raw instrument files were converted to mzXML [26] and extracted using the two-stage hybrid feature detection and alignment procedure available in apLCMS [27] using five parameter settings optimized for a range of peak intensities. The resulting feature tables were merged using xMSanalyzer [28] and batch-corrected using ComBat [29]. Metabolites were identified by comparing detected m/z and retention time to an in-house database of 1,200 standards analyzed using the same method parameters that included a wide range of environmental and endogenous compounds. Metabolite identifications were determined by matching mass-to-charge ratio (m/z) and retention time with a tolerance of 5ppm and 15 s, respectively. Metabolic features were uniquely defined by their m/z, retention time, and ion intensity.

### Data processing and statistical analysis

Before data processing and statistical analysis, the overall composition of identified metabolites was plotted. We followed a previously published urine-based metabolomics pipeline to adjust for individual hydration using specific gravity measurement and remove unwanted variation in this analysis [30]. We restricted all analysis to metabolites annotated with level 1 confidence [31, 32] with confirmed structures against reference standards and those present in at least 75% of the samples. These metabolites then underwent missing value imputation and further analyses. Missing values in metabolomics studies can arise due to values being below the detection limit and/or the absence of the metabolite [33]. Missing values on ion intensity for metabolites that went into analysis were imputed using a random forest algorithm as implemented in the R package missForest [34, 35]. All ion intensities of metabolites were log10-transformed and standardized to stabilize variation and meet linear model assumptions. In addition, to recover significant metabolites from those excluded, we conducted a non-parametric Wilcoxon test on each of these metabolites to compare their ion intensities between postfire and baseline and presented the results graphically (Supplemental Table 2; Supplemental Fig. 3).

Considering potential disadvantages of uneven cluster sizes [36], and to facilitate direct comparison of results between firefighters responding to WUI and MSF (where a 1:1 matched sampling scheme by participant was implemented [37]), we included 1:1 matched baseline-postfire urine sample pairs from firefighters responding to WUI fires for regression analysis. To identify differential metabolites by fire exposure, regression analysis was performed using complete cases. To clarify, “differential” in this analysis refers to metabolites that significantly changed in expression levels between baseline and post-fire samples within each fire type. For WUI samples, a linear mixed effects model with random intercept for each firefighter was fitted for each urinary feature with preprocessed ion intensity being the response and fire exposure status (baseline vs. post-fire) being the main predictor, while adjusting for covariates including age, years of experience, Hispanic ethnicity, and rank at study enrollment. Ethnicity was categorized as non-Hispanic White, Black, Hispanic, or Other. For the purposes of this analysis, ethnicity was dichotomized into Hispanic and non-Hispanic to align with prior research demonstrating biologically meaningful differences between these groups [30, 38]. The same model was fitted on MSF samples. We adjusted for multiple testing by controlling the family-wise error rate at the false discovery rate (FDR) 0.05 level, and we defined differential metabolites as those with FDR q < 0.05. The differential status was presented and compared both graphically and in tabular format. All preprocessing and statistical analyses were performed in the R programming environment (version 4.3.2) [39].

### Pathway analysis

We performed enrichment analysis for each chromatography-ESI mode to investigate changes in metabolic profiles associated with WUI and MSF exposure at the metabolic pathway level. Enrichment analysis was conducted using metabolite-set enrichment analysis (MSEA) as implemented on MetaboAnalyst (version 6.0) [40]. Specifically, all annotated metabolites were included as the reference set, and all differential metabolites at the FDR 0.05 level were included as the metabolites of interest. The Kyoto Encyclopedia of Genes and Genomes (KEGG) pathways [41] were utilized as the pathway sets. To reduce false positive metabolite matches, we restricted interpretation to pathways significantly enriched with at least three metabolites. To minimize false positives in enriched pathways, we applied Holm-Bonferroni adjustment for multiple testing. We defined enriched pathways as those with an adjusted p-value less than 0.05. Pathway enrichment status was presented and compared in a dot plot facetted by study/fire type. All pathways with raw p-values less than 0.05 were included in the graphical comparison.

In addition to evaluating differential and enrichment status by WUI fire exposure, we also performed differential analysis and pathway analysis for MSF exposure and compared the number of differentially expressed metabolites and the patterns of metabolic enrichment by fire exposure between the two fire types.

## Results

### Study population and sample

A total of 87 WUI firefighters contributed 204 urine samples. This group included only one female firefighter, and it also included one male firefighter with only one sample available. We excluded these 2 participants and their corresponding urine samples from statistical analyses, resulting in a final group of 85 male firefighters who contributed 85 urine samples from baseline visits and 117 urine samples from post-fire visits. Given that post-fire samples included repeated samples from some participants following different fire incidents, each baseline sample was paired with the closest post-fire sample in time by participant, resulting in 85 pairs of baseline-postfire samples for WUI firefighters. In the MSF study, 100 male firefighters donated a total of 200 urine samples. Among them, one firefighter had only one visit available, and one had missing demographic information. We excluded samples from these two participants from analyses and paired each baseline sample with the closest post-fire sample in time by participant, resulting in 98 firefighters responding to MSF contributing 98 pairs of baseline-postfire samples.

Firefighters responding to WUI fires had an average age (standard deviation) of 37.1 (10.0) years and an average of 12.2 (9.7) years in firefighting service. 34% of the firefighters responding to WUI fires were Hispanic, and more than one-third were in the firefighter/paramedic/emergency medical technician (EMT) category. Nearly 30% of firefighters responding to WUI fires held college degrees, and more than 78% had a history of smoking (defined as having ever smoked at least 100 cigarettes). In comparison, firefighters responding to MSF had an average age of 37.5 (8.6) years and an average of 9.0 (6.9) years in firefighting service. 19% of MSF firefighters were Hispanic, 60% were in the firefighter/paramedic/emergency medical technician (EMT) category, and more than 78% had a history of smoking. We included education and smoking status in Table 1 to demonstrate that the WUI and MSF firefighter groups were generally similar in key demographic and lifestyle characteristics. Although not used as confounders in subsequent analyses, these variables help contextualize the comparability between groups. Overall, firefighters responding to WUI fires and MSF were comparable in age, education, and smoking history. However, firefighters responding to WUI fires had more firefighting experience compared to firefighters responding to MSF.

**Table 1:** Summary statistics of demographics for male firefighters responding to WUI fires (*N* = 85) for the wildland-urban-interface firefighter study and male firefighters responding to MSF (*N* = 98) for the structure fire study

	WUI (*N* = 85)	MSF (*N* = 98)
Age	37.1 (10.0)	37.5 (8.6)
Time in Firefighting Service (years)	12.2 (9.7)	9.0 (6.9)
Hispanic Ethnicity		
No	56 (65.9%)	79 (80.6%)
Yes	29 (34.1%)	19 (19.4%)
Rank at Sampling		
Captain/Chief/Engineer	21 (24.7%)	40 (40.8%)
Firefighter/Paramedic/EMT^a^	37 (43.5%)	58 (59.2%)
Driver Operator	15 (17.6%)	
Other	12 (14.1%)	
Education		
No. Missing value	0	12
College graduate and higher	26 (30.6%)	30 (34.9%)
Some College or lower	59 (69.4%)	56 (65.1%)
Ever Smoked 100 Cigarettes		
No. Missing value	30	14
Yes	43 (78.2%)	66 (78.6%)
No	12 (21.8%)	18 (21.4%)

^a^EMT, emergency medical technician

### High-resolution metabolomics

Using targeted, high-resolution metabolomics combined with an in-house library of approximately 1,200 environmental pollutants, we detected 244, 297, 320, and 266 urinary metabolites with level 1 annotation confidence in HILIC(-), HILIC(+), C18(-), and C18(+) modes, respectively. These metabolites predominantly consisted of endogenous compounds, including carnitine, bile acids, fatty acids, sterols, and steroid hormones, as well as biomarkers of exogenous chemicals such as pesticides, PFAS, phenols, phthalates, and smoking-related compounds (Supplemental Fig. 1). After excluding metabolites present in less than 75% of urine samples, we retained a total of 179, 255, 262, and 213 differential metabolites from WUI samples, respectively. Similarly, we retained 173, 257, 268, and 215 metabolites from MSF samples. Out of the 218 excluded metabolites, 28 showed significant changes after WUI fire exposure based on a raw p-value of 0.05. However, only 10-methyloctadecanoic acid remained statistically significant at an FDR level of 0.05, likely due to skewness in the post-fire distribution (Supplemental Fig. 3 A & 3B). A comprehensive list of excluded metabolites and details of the statistical tests can be found in Supplementary Table 2.

### Differential analysis

**Fig. 1:**
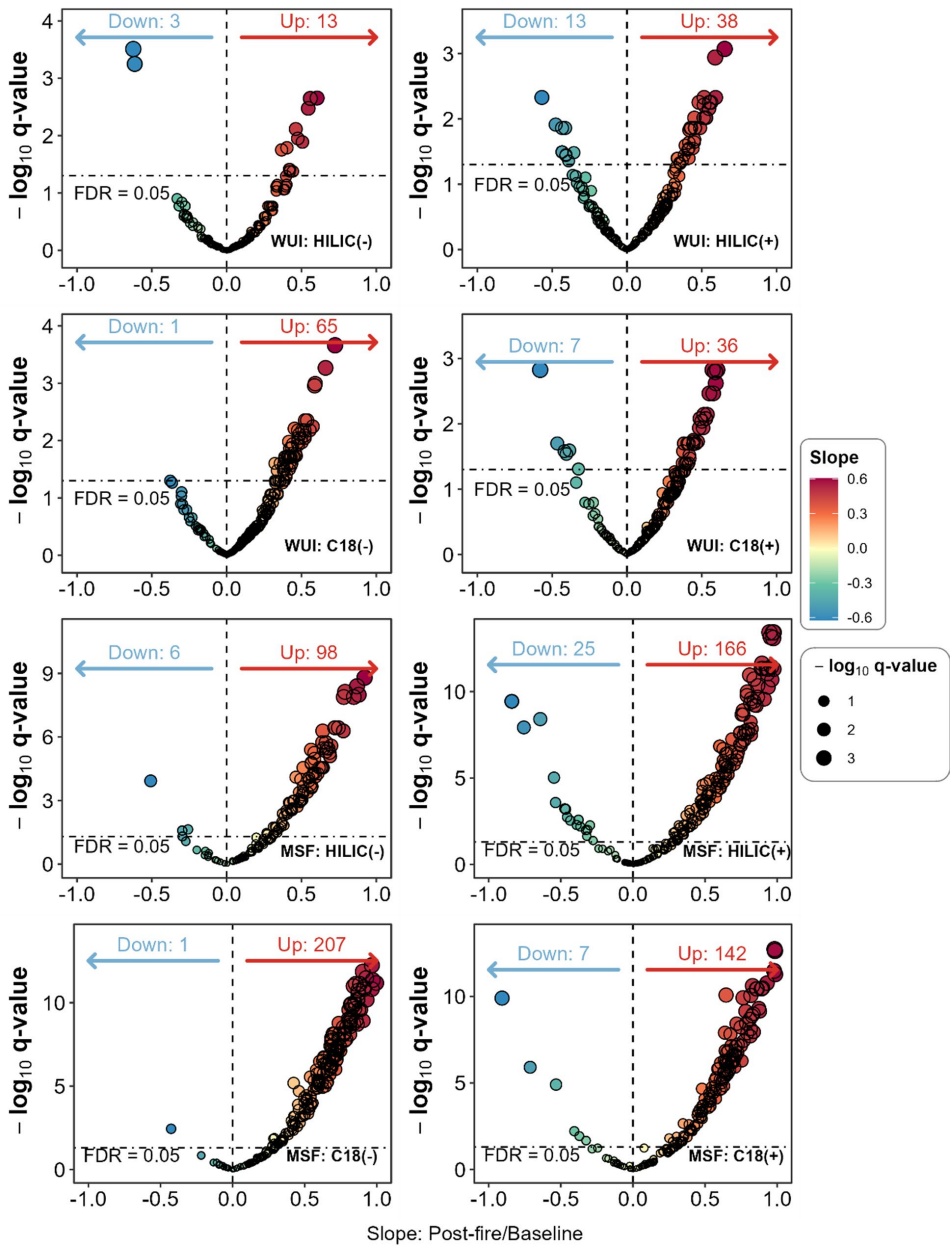
Volcano plot of differential metabolites comparing baseline urine samples to post-fire urine samples from male firefighters responding to WUI fires and MSF. The top 4 subplots are for WUI fires whereas the rest are for MSF. Within each subplot, the comparison groups are baseline and postfire levels of metabolites for a fire type. The slope term was from the main model where exposure status (postfire/baseline) was the main predictor and preprocessed ion intensity was the response. Upregulation is marked in red whereas downregulation in light blue. The horizontal dashed line marked “FDR = 0.05” is the boundary that served as the threshold for differential status in this study. FDR q-values were -log_10_ transformed

After adjustment for age, Hispanic ethnicity, time in fire service, and rank, the main model identified 16, 51, 66, and 43 differential metabolites for HILIC(-), HILIC(+), C18(-), and C18(+) modes, respectively, when comparing baseline and postfire urine samples of firefighters responding to WUI fires (Fig. 1). Subsequently, after adjusting for the same set of covariates, our main model identified 104, 191, 208, and 149 differential metabolites for HILIC(-), HILIC(+), C18(-), and C18(+) modes, respectively, from urine samples of firefighters responding to MSF (Fig. 1).

A comprehensive list of metabolites from firefighters responding to WUI fires and MSF, including model details, is available in Supplemental Table 1. Overall, we observed a greater number of upregulated than downregulated metabolites in both WUI and MSF samples (Fig. 1). Specifically for firefighters responding to WUI fires, all 16 differential metabolites identified in HILIC(-) mode were endogenous compounds. Out of the 51 differential metabolites identified in HILIC(+) mode, five were markers of pesticides and the remainder were endogenous compounds. In C18(-) mode, only one phthalate was differentially expressed, with the rest being endogenous metabolites. In C18(+) mode, two pesticides, two PFAS species, and one tobacco use biomarker were differentially expressed. For firefighters responding to MSF, HILIC(-) identified five differentially expressed metabolites associated with pesticides; HILIC(+) identified 17 pesticide metabolites; C18(-) identified one marker for a PFAS, one for a pesticide, and one for phenol; C18(+) identified two markers for PFAS and five for pesticides, with the rest coming from endogenous classes. The top ten differential metabolites for WUI fire exposure by slope for each mode are shown in Table 2 along with the same metabolites from MSF exposure. The comparison between WUI and MSF exposure revealed a total of 148 differentially expressed metabolites common to both studies and 27 unique metabolites specific to WUI fires. These unique urinary metabolites consisted mostly of endogenous molecules, along with one marker for a phthalate (monoisnonyl phthalate) and one tobacco use biomarker (nicotine).

**Table 2:** Top 10 differential metabolites by slope within each mode comparing baseline to post-fire urine samples with FDR < 0.05 for firefighters responding to WUI fire exposure in comparison with MSF MZ: mass to charge ratio, RT: retention time in seconds, slope: the coefficient estimates for the exposure status (baseline-postfire: 0–1) term of the main model. Positive slopes indicated an increase in the ion intensity of metabolites after fire exposure whereas negative slopes indicated a decrease

						WUI			MSF		
Mode	CID	Metabolite	Formula	MZ	RT	Slope	*P*-value	FDR	Slope	*P*-value	FDR
C18(+)	70,679,121	Octenoyl-L-carnitine	C25H47NO5	286.2013	206.8503	0.60	< 0.001	0.001	0.63	< 0.001	< 0.001
C18(+)	92,136	2-Aminoadipic acid	C6H11NO4	162.0761	24.0290	0.59	< 0.001	0.002	0.53	< 0.001	< 0.001
C18(+)	151,730	5-Hydroxypipecolic acid	C6H11NO3	146.0812	22.9124	0.59	< 0.001	0.002	0.48	< 0.001	< 0.001
C18(+)	18,189	4-Acetamidobutyric acid	C6H11NO3	146.0812	29.8209	0.59	< 0.001	0.002	0.48	< 0.001	< 0.001
C18(+)	774	Histamine	C5H9N3	112.0869	17.4500	0.58	< 0.001	0.003	0.58	< 0.001	< 0.001
C18(+)	90,659,885	Hydroxybutyrylcarnitine	C11H21NO5	248.1492	19.7800	0.57	< 0.001	0.001			
C18(+)	2,769,669	1 H,1 H-Perfluoropentylamine	C5H4F9N	250.0273	80.4000	0.55	< 0.001	0.003	0.40	0.003	0.005
C18(+)	439,176	Methylthioadenosine	C11H15N5O3S	298.0968	31.2521	0.54	< 0.001	0.007	0.88	< 0.001	< 0.001
C18(+)	10,917	L-Carnitine	C7H15NO3	162.1125	22.1876	0.53	< 0.001	0.008			
C18(+)	2,724,480	L-Cartinine	C7H15NO3	162.1125	18.7000	0.53	< 0.001	0.008			
C18(-)	5,312,738	[C10.1]−10-hydroxy-2-decenoic acid	C10H18O3	185.1183	55.1000	0.72	< 0.001	< 0.001			
C18(-)	31,401	Ursodeoxycholate	C24H40O4	391.2854	229.4000	0.66	< 0.001	0.001	0.51	< 0.001	< 0.001
C18(-)	5,282,713	[C8.1]−2-Octenoic acid	C8H14O2	141.0921	161.4000	0.59	< 0.001	0.001	0.65	< 0.001	< 0.001
C18(-)	92,136	Aminoadipate	C6H11NO4	160.0615	18.9000	0.59	< 0.001	0.001	0.81	< 0.001	< 0.001
C18(-)	110,394	Monoisononyl phthalate	C17H24O4	291.1602	230.7300	0.58	< 0.001	0.006			
C18(-)	1028	Prephenic acid	C10H10O6	225.0405	18.3000	0.55	< 0.001	0.007	0.50	< 0.001	0.001
C18(-)	464	Hippurate	C9H9NO3	178.0510	28.0000	0.54	< 0.001	0.004	0.72	< 0.001	< 0.001
C18(-)	5960	Aspartate	C4H7NO4	132.0302	18.9000	0.52	< 0.001	0.004	0.74	< 0.001	< 0.001
C18(-)	18,189	4-Acetamidobutanoate	C6H11NO3	144.0666	19.4000	0.52	< 0.001	0.009			
C18(-)	10,972	N-Acetylglycine	C4H7NO3	116.0353	19.7000	0.52	< 0.001	0.008	0.72	< 0.001	< 0.001
HILIC(+)	16,247	Aminocarb	C11H16N2O2	209.1285	208.2000	0.65	< 0.001	0.001	0.64	< 0.001	< 0.001
HILIC(+)	5,280,933	Gamma-Linolete	C18H30O2	279.2319	66.0000	0.59	< 0.001	0.005	0.50	< 0.001	0.001
HILIC(+)	150,923	3-Methyl-2-Oxindole	C9H9NO	148.0757	70.3000	0.59	< 0.001	0.001	0.58	< 0.001	< 0.001
HILIC(+)	10,917	L-Carnitine	C7H15NO3	162.1125	294.3000	0.56	< 0.001	0.006			
HILIC(+)	68,570	Estradiol-17Alpha	C18H24O2	137.0961	68.2000	0.55	< 0.001	0.006	0.93	< 0.001	< 0.001
HILIC(+)	736,715	Urocate	C6H6N2O2	139.0502	267.8000	0.55	< 0.001	0.007	0.65	< 0.001	< 0.001
HILIC(+)	6,426,851	Isovaleryl-L-carnitine	C12H23NO4	246.1700	230.2000	0.53	< 0.001	0.010	0.35	0.003	0.005
HILIC(+)	53,481,619	Valeryl-L-carnitine	C12H23NO4	246.1700	230.2000	0.53	< 0.001	0.010	0.35	0.003	0.005
HILIC(+)	6,426,901	2-Methylbutyroylcarnitine	C12H23NO4	246.1700	232.7000	0.53	< 0.001	0.010	0.35	0.003	0.005
HILIC(+)	439,227	Pipecolate	C6H11NO2	130.0863	265.8000	0.52	< 0.001	0.005			
HILIC(-)	8094	Heptanoate	C7H14O2	129.0921	66.7000	0.60	< 0.001	0.002	0.54	< 0.001	< 0.001
HILIC(-)	26,612	[C10.0]−10-Hydroxydecanoic acid	C10H20O3	187.1340	74.1000	0.56	< 0.001	0.002	0.44	0.001	0.003
HILIC(-)	74,300	10-Hydroxydecanoate	C10H20O3	187.1340	83.4085	0.56	< 0.001	0.002	0.44	0.001	0.003
HILIC(-)	8892	Hexanoate	C6H12O2	115.0765	78.3100	0.55	< 0.001	0.003	0.48	0.001	0.002
HILIC(-)	61,743	[C10.1]−9-Decenoic acid	C10H18O2	169.1234	59.3000	0.51	< 0.001	0.013	0.50	< 0.001	0.001
HILIC(-)	95,433	[2-OH,2-Me-4.0]−2-Hydroxy-2-methylbutyric acid	C5H10O3	117.0557	144.0786	0.48	< 0.001	0.011	0.59	< 0.001	< 0.001
HILIC(-)	5,282,713	[C8.1]−2-Octenoic acid	C8H14O2	141.0921	68.0000	0.46	< 0.001	0.008	0.56	< 0.001	< 0.001
HILIC(-)	18,189	4-Acetamidobutanoate	C6H11NO3	144.0666	273.0341	0.44	0.004	0.042	0.37	0.009	0.017
HILIC(-)	26,613	[C8.0]−3-Hydroxyoctanoic acid	C8H16O3	159.1027	72.1000	0.42	0.003	0.038			
HILIC(-)	971	Oxalate	C2H2O4	88.9880	343.2486	0.42	0.004	0.042	0.41	0.002	0.004

### Pathway analysis

**Fig. 2:**
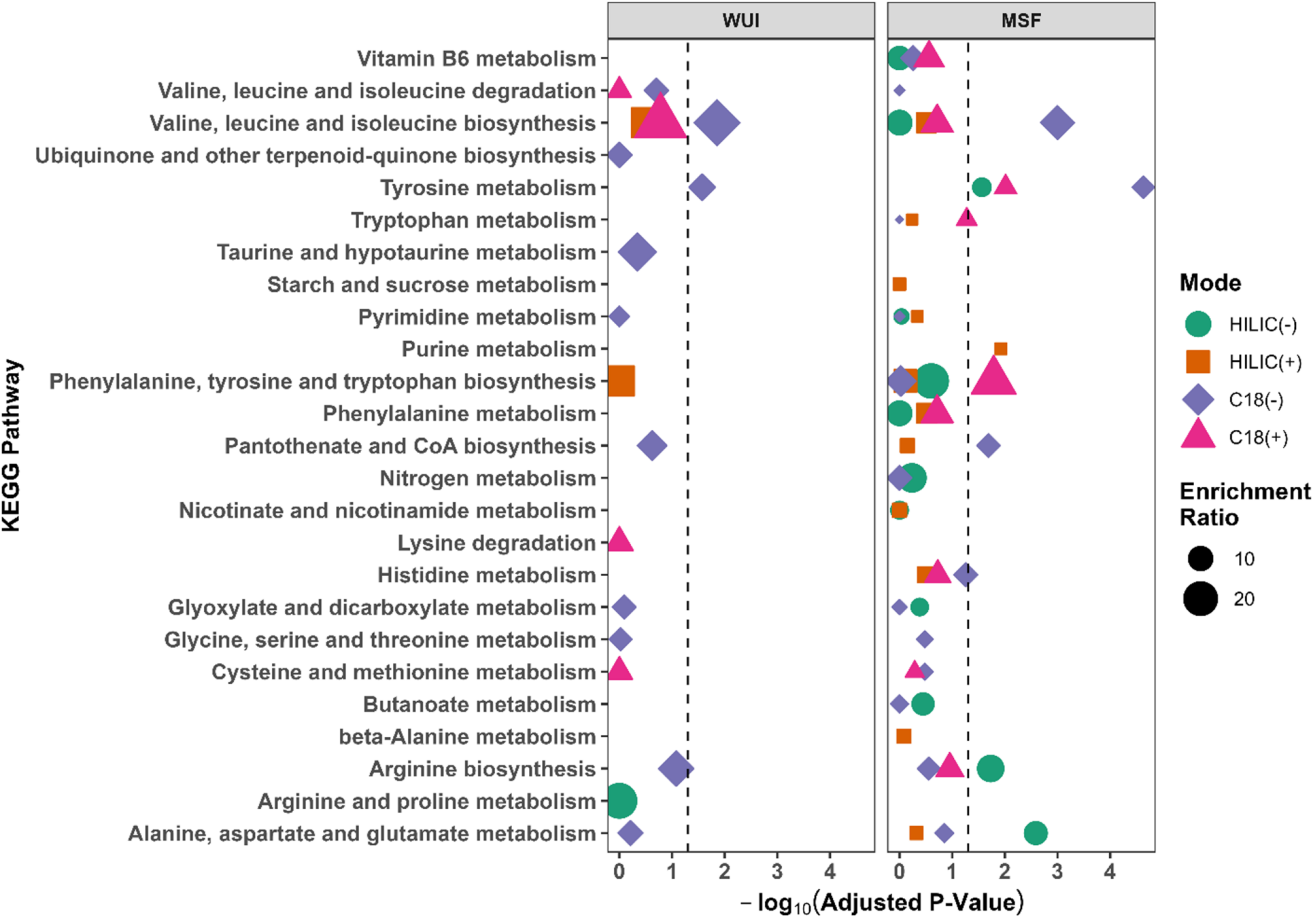
Metabolic enrichment plot for the comparison between baseline vs. post-fire urine samples among male firefighters responding to WUI fires & MSF, by four separation-ESI modes. Statistical significance was determined by Fisher’s Exact test. Multiple testing was adjusted using Bonferroni’s method to avoid false positives. The enrichment ratio was defined as the ratio of the number of significant hits from the user input list of differential metabolites to the number of expected metabolites in each pathway. Separation-ESI is marked in green, orange, purple, and pink. The enrichment ratio is reflected by the size of the dot where large sizes indicate more enrichment. The adjusted P-values were -log_10_ transformed

Upon restricting the size of pathways to contain a minimum of three metabolites and a raw p-value of 0.05 or lower, the pathway analysis yielded a total of 18 enriched pathways for WUI fires, distributed as follows: 1 pathway in HILIC(-) mode, 2 pathways in HILIC(+) mode, 11 pathways in C18(-) mode, and 4 pathways in C18(+) mode. For MSF, the pathway analysis identified 49 enriched pathways including 12 pathways in HILIC(-) and HILIC(+) mode each, 16 in C18(-) mode, and 9 in C18(+) mode. After adjustment for multiple testing using Bonferroni’s method at 0.05 level, 2 metabolic pathways were reported for WUI fires including valine, leucine and isoleucine biosynthesis and tyrosine metabolism. In contrast, 9 pathways were significantly enriched after MSF exposure including biosynthesis of valine, leucine, isoleucine, phenylalanine, tyrosine, tryptophan, pantothenate and CoA, and arginine, and metabolism of tyrosine, alanine, aspartate, glutamate and purine (Fig. 2; Supplemental Table 3). Perturbations in the biosynthesis of phenylalanine, tyrosine, tryptophan, and pantothenate and CoA were also reported after WUI fires, though these did not reach statistical significance. Metabolism and biosynthesis of tyrosine, tryptophan, and phenylalanine were significantly disturbed only in post-fire samples from firefighters responding to MSF, not after WUI fires. Valine, leucine and isoleucine biosynthesis and tyrosine metabolism were significantly enriched following both types of fires at the adjusted *p* < 0.05 level. MSF exposure introduced more significantly disturbed pathways. Notably, the metabolism of purine, alanine, aspartate, and glutamate, and biosynthesis of phenylalanine, tyrosine, tryptophan, and pantothenate and CoA, were perturbed only in samples from MSF firefighters at the adjusted *p* < 0.05 level.

## Discussion

Our comparison of the urinary metabolomes of firefighters responding to WUI fires and MSF before and after fire exposure revealed both shared and unique alterations in metabolites and metabolic processes for each fire exposure. For both exposures, metabolites predominantly included endogenous molecules such as carnitine, bile acids, fatty acids, sterols, steroid hormones, as well as biomarkers of environmental chemicals such as pesticides, PFAS, phenols, and phthalates. These metabolites were involved in a wide spectrum of metabolic processes, and we observed enrichment in numerous metabolic pathways, including valine, leucine, and isoleucine biosynthesis and tyrosine metabolism, among others. Notably, valine, leucine, and isoleucine biosynthesis and tyrosine metabolism were perturbed after both types of fires. These findings suggested that like MSF, exposure to WUI fires may introduce significant physiological stress in firefighters, potentially leading to long-term health implications. Additionally, while both types of fires caused metabolic disturbances, the specific pathways enriched in response to individual fires differed, likely reflecting the unique exposures of the fire events.

### WUI fire exposure & health risks

We detected both endogenous molecules and exogenous chemicals in WUI urine samples, with most differential metabolites coming from the endogenous class. Specifically, out of the 176 differential metabolites, 11 were environmental chemicals including pesticides, PFAS, phthalates, and tobacco related compounds. Possible sources of these chemicals include ash, soot, firefighting foam and toxic smoke [42], and lifestyle factors that relate to tobacco use. Beyond chemical hazards, firefighters attending WUI fires face significant physical challenges. Heat stress, combined with physical exertion, could be another significant exposure for firefighters, as it not only intensifies muscle fatigue but also poses a risk to the cardiovascular system [43]. Therefore, the interplay between chemical and physical stressors likely contributed to the observed changes in metabolic profiles and processes, a dynamic that warrants further investigation.

In this study, we identified various metabolites whose levels were significantly altered after fire exposure. The metabolites discussed here were chosen based on their biological relevance to metabolic processes affected by oxidative stress and inflammation, as well as their statistical significance in our analysis. We focused on those that play critical roles in energy production, immune response, and oxidative stress, which are central to the physiological impact firefighters experience during fire exposure as well as the development of cancer.

### Amino acid metabolism and oxidative stress

Two pathways were significantly enriched after both WUI and MSF responses, including valine, leucine, isoleucine biosynthesis, and tyrosine metabolism. Valine, leucine, and isoleucine (Supplemental Table 1, Supplemental Fig. 2) are essential amino acids (branched-chain amino acids, BCAAs) that are obtained via external sources and are crucial for protein synthesis and energy production. Firefighting is physically demanding which may lead to increased dietary intake of BCAAs via energy drinks or nutritional supplements, leading to enrichment in metabolism of essential amino acids. Daskalaki et al. (2015) reported exercise-induced metabolic alterations involving significant changes in amino acid oxidation, purine metabolism, and tryptophan metabolism [44]. Another study also found that levels of creatinine, lactate, pyruvate, alanine, β-hydroxybutyrate, acetate, and hypoxanthine increased following short-term exercise [45]. Persistent elevation in circulating BCAAs can lead to elevated cardiovascular risks over time [46].

Tyrosine is a non-essential amino acid that can be synthesized from phenylalanine and plays several important roles including in protein synthesis and as a precursor for neurotransmitters [47]. Disruption of tyrosine metabolism is linked to various conditions, including several types of cancer, such as gastroesophageal malignancies [48] and lung cancer [49]. However, these conditions are associated with a decreased level of tyrosine.

The arginine biosynthesis pathway was enriched in MSF post-fire samples compared to baseline. Arginine biosynthesis was also potentially disrupted in patients with high oxidative stress [50]. Serine was elevated after WUI fire exposure (Supplemental Fig. 2). Serine contributes to cellular metabolism largely through refueling one-carbon (1 C) metabolism which provides 1 C units (methyl groups) that are required for methylation processes including DNA methylation and protein post-translational modification [51]. Serine also plays a vital role in maintaining metabolic homeostasis and health in stress situations [52]. Additionally, cancer cells require 1 C units for high proliferation and elevated levels of serine advantages tumors and drives oncogenesis [51, 53]. Elevated level of serine post WUI fire exposure (C18 mode; Supplemental Table 1, Supplemental Fig. 2) may indicate cellular repair triggered by oxidative stress from fire exposure. Chronic dysregulation of serine metabolism and disruptions in one-carbon metabolism might affect genomic stability and impair various cellular functions, potentially leading to long-term health consequences such as increased cancer risk. We previously identified enrichment in glycine, serine, and threonine metabolism when comparing post-fire to baseline urine samples from firefighters responding to MSF analyzed in HILIC and C18 mode, though it did not reach statistical significance [37]. Similarly, our re-analysis of MSF urine samples reported this pathway, but it remained statistically nonsignificant, after limiting analysis to level 1 metabolites.

Observed enrichments in both essential and non-essential amino acids likely reflect a complex set of responses to WUI fire exposures, potentially involving altered energy production and antioxidant responses in firefighters. Chronic disturbance in these critical pathways can cause elevated risks of cardiovascular conditions and increased oxidative and inflammatory burden that are closely related to cancer development and progression [12, 13], which may help explain the elevated cancer risks of firefighters.

### Metabolism of vitamins and lipids, and disruption of energy production, oxidative stress

Pantothenate and CoA biosynthesis were enriched after experiencing WUI fires and significantly enriched after MSF exposure. Pantothenate is a vitamin that is essential for the synthesis of coenzyme A (CoA), which is crucial for numerous metabolic processes, including the citric acid cycle for energy production, fatty acid synthesis and oxidation, and lipid synthesis. Upregulation of CoA also occurs in response to cellular oxidative stress [54].

Ubiquinone (coenzyme Q) is a crucial component for electron transport and ATP generation whose deficiency may cause conditions such as type 2 diabetes and cardiovascular disease [55, 56]. Terpenoid-quinones are derived from terpenoids and quinones, which are important for electron transport and protection against oxidant stress [57, 58]. Homogentisate is an integral compound in the ubiquinone and other terpenoid-quinone biosynthesis process and was elevated postfire (Supplemental Table 1, Supplemental Fig. 2), possibly indicative of cellular repair induced by oxidative stress from WUI fire exposures. Chronic fire exposure and resulting metabolic perturbation could lead to disruption in energy production and persist oxidative stress in firefighters.

Overall, the enrichment patterns observed indicated significant metabolic disturbances following WUI fire exposure. These changes reflect increased physical and physiological stress, leading to disruptions in metabolism of amino acids, lipids, and vitamins, which are likely to relate to energy production and oxidative response due to firefighting. Chronic disruptions of these metabolic processes can result in an elevated risk of cardiovascular and neurological conditions, oxidative and inflammatory burdens, genomic instability, and cancer [12, 13, 46, 59]. A global metabolic profiling study comparing tumor and paired nontumor samples revealed a set of 207 common to liver, pancreas and breast tumor specimens where most of the shared set belonged to lipid and amino acid classes [60]. Several of the pathways we identified as enriched in firefighters, such as valine, leucine, and isoleucine biosynthesis, as well as tyrosine metabolism, are also elevated in tumor specimens [60]. This overlap further supports the conclusion that firefighters responding to WUI fires may face an increased risk of cancer.

### WUI-MSF comparison

We detected markers for endogenous and exogenous chemicals in MSF urine samples, before and after exposure. Out of a total of 652 differential metabolites, 32 were environmental chemicals. Overall, MSF exposure resulted in a greater number of differential metabolites compared to WUI fires. Consequently, a wider range of metabolic processes were found to be enriched.

In addition to the shared enrichment with WUI, MSF exposure introduced a broader set of disturbances to metabolic processes including metabolism of purine, arginine, aspartate, glutamate, alanine, phenylalanine, and tryptophan. Purines are critical to nucleic acid synthesis [61]. Arginine plays an important role in detoxification of ammonia [62] and has been associated with increased oxidative stress [50]. Aspartate regulates nucleotide synthesis and serves as a precursor for four essential amino acids [63]. Glutamate is the most abundant excitatory neurotransmitter in the central nervous system (CNS) and is involved in various cognitive functions [64]. L-alanine plays various important roles in the body including in protein synthesis and the functioning of the immune system, as well as an energy source for intense exercise [65]. Phenylalanine is a building block of other amino acids, and a precursor of tyrosine and dopamine [66]. The enrichment of phenylalanine and tyrosine metabolism, indicative of an anti-oxidative response to fire-related tasks and chronic stress, may lead to cognitive impairment and an increased risk of several types of cancers [48, 49]. Tryptophan is an essential amino acid that serves as a precursor for serotonin and the kynurenine pathway, which are crucial for mood regulation, immune response, and cognitive function [67], and whose metabolism has been reported to be enriched in tumor specimens from breast and pancreas cancer tissue [60]. MSF postfire samples had elevated levels of tryptophan, kynurenic acid, and serotonin. We previously reported increased levels of tryptophan and tryptophan derivatives such as kynurenic acid after MSF [37]. Other studies have also reported altered expression of tryptophan associated with air pollution [68], which may introduce the same stressors as those experienced by firefighters during MSF tasks. The metabolic pathways disrupted by MSF exposure indicate potential oxidative stress and disturbances in cognitive functions and potentially help explain elevated cancer risks in firefighters.

The number of shared differential metabolites following WUI fires compared to MSF alone indicates both common and unique metabolic perturbations. Both WUI and MSF exposure induced changes in valine, leucine, isoleucine, and tyrosine, demonstrating shared metabolic perturbations in metabolism of amino acids. WUI fires affected pathways related to valine, leucine, isoleucine, and tyrosine biosynthesis while MSF uniquely impacted the metabolism or biosynthesis of purine, phenylalanine, tryptophan, arginine, alanine, aspartate, and glutamate. The WUI pathways suggest impacts on energy production and oxidative stress, whereas the higher number of differential metabolites and unique pathways from MSF suggest a more pronounced disruption of neurotransmitters and DNA/RNA synthesis, in addition to altered energy production and oxidative stress. Both types of fires may present significant health risks due to perturbations in critical metabolic processes. Overall, MSF resulted in more extensive alterations in both individual metabolites and metabolic processes compared to WUI fires, which may contribute to a wider range of health concerns. Longitudinal or targeted studies are needed to evaluate the associations of urine metabolites with changes in other markers of cancer risk, such as epigenetic changes, and ultimately cancer incidence.

### Strengths and limitations

The application of high-throughput metabolomics enabled simultaneous detection of numerous metabolites. This capability was further enhanced by a comprehensive in-house library of approximately 1,200 environmental chemicals and endogenous molecules, thereby strengthening our confidence in the interpretation of results. The 1:1 matched baseline-postfire analytical approach enabled robust statistical analysis, enhanced the ability to detect significant signals, and inherently controlled for time-invariant confounding factors. However, this study was relatively short-term, and we could not account for behavioral changes, such as beverage and medication use between baseline and postfire sampling, or post-fire environmental exposures that can affect firefighters’ exposure other than firefighting [69, 70]. Also, this study ended up limited with male firefighters. The comparison between WUI and MSF was intended to be exploratory rather than strictly statistical, given several limitations-most notably, particularly given the differences in PPE/safety practices, as well as differences in participant characteristics among others. While all participants in this study were employed in the southwestern United States, we were unable to account for potential differences in the structural components of fire exposures across MSF and WUI settings. Variability in building materials. such as older versus newer construction, or differences in insulation, roofing, and synthetic content, could influence the chemical composition of smoke and, consequently, the metabolites detected in urine. Incorporating more detailed data on the types, age, and materials of structures involved in fire responses could enhance the interpretability and specificity of metabolomic signatures. Future longitudinal and targeted studies may consider expanding to include samples from female firefighters and addressing the limitations by providing a more robust framework for analyzing temporal changes and reducing variability.

## Conclusions

This study addresses potential mechanisms of cancer and health risks faced by WUI firefighters. By leveraging high-throughput metabolomics, we explored metabolic alterations in urinary metabolites resulting from exposure to WUI fires and compared them with MSF. Relationships with changes in metabolites and potential mechanisms of cancer and other health risks were suggested. WUI fires expose firefighters to significant physiological stresses, which disturb a broad range of metabolic processes and present the potential for substantial long-term health implications. Our analysis also revealed that WUI and MSF exposure induced similar metabolic perturbations while each type of fire also showed unique metabolic disruptions. Our findings of altered metabolites provide insights into the endogenous and exogenous chemical exposures faced by firefighters. This research also underscores the need for further investigations into long-term health implications, including cancer risks, associated with these occupational hazards. Such work can inform targeted interventions to mitigate these risks.

## Supplementary Information

Supplementary Material 1

Supplementary Material 2

## Data Availability

Given privacy concerns, data is available upon request.
