# Supplementary material for: Evaluating urinary metabolic profiles with wildland-urban-interface (wui) fire exposure among male firefighters: a comparison with municipal structure fires (msf)
Source: Environ Health. 2025 Nov 17;24:88. doi: 10.1186/s12940-025-01239-7 (PMC12625405; doi:10.1186/s12940-025-01239-7)
Supplement: Supplementary file 1 — Supplementary Material 1 [file 12940_2025_1239_MOESM1_ESM.docx]

Evaluating Urinary Metabolic Profiles with Wildland-Urban-Interface (WUI) Fire Exposure among Male Firefighters: A Comparison with Municipal Structure Fires (MSF)

Tuo Liu^1^, Melissa A. Furlong^1^, Justin M. Snider^3^, Shawn Beitel^1^, Catherine E. Mullins^4^, Douglas I. Walker^4^, Jaclyn M. Goodrich^5^, Derek J. Urwin^6,7^, Jamie Gabriel^7^, Jeff Hughes^8^, John J. Gulotta^9^, Miriam M Calkins^10^, Yiwen Liu^2^, Frank A. von Hippel^1^, Paloma Beamer^1^, Jefferey L. Burgess^1^

^1^Department of Community, Environment, and Policy, Mel and Enid Zuckerman College of Public Health, University of Arizona, Tucson, US

^2^Department of Epidemiology and Biostatistics, Mel and Enid Zuckerman College of Public Health, University of Arizona, Tucson, US

^3^School of Nutritional Sciences and Wellness, University of Arizona, Tucson, US

^4^Gangarosa Department of Environmental Health, Rollins School of Public Health, Emory University, Atlanta, GA

^5^Department of Environmental Health Sciences, School of Public Health, University of Michigan, Ann Arbor, MI

^6^Chemistry and Biochemistry Department, University of California at Los Angeles, Los Angeles, CA

^7^Los Angeles County Fire Department, Los Angeles, CA

^8^Orange County Professional Firefighters Association, Tustin, CA

^9^Tucson Fire Department, Tucson, AZ

^10^National Institute for Occupational Safety and Health, Centers for Disease Control and Prevention, Cincinnati, OH, USA

Corresponding author [mfurlong@arizona.edu](mailto:mfurlong@arizona.edu), 1295 N Martin Ave, Tucson AZ 85724

Supplemental Figure 1. Composition tree map of level-1 metabolites identified in firefighters' urine sample from WUI & MSF by separation-ESI model.


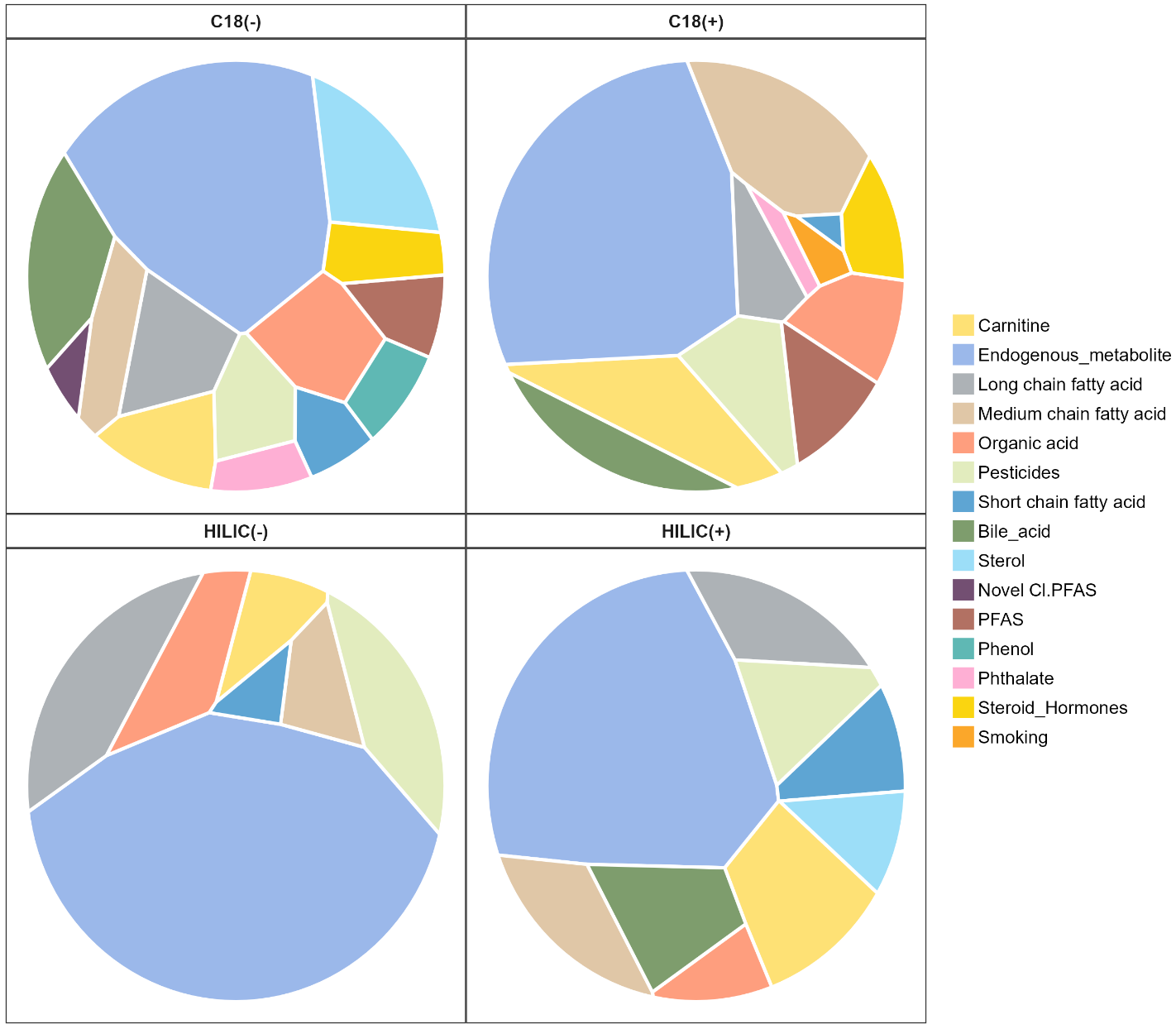


Supplemental Figure 2A. Violin plots of select metabolites involved in enriched pathways following WUI fire exposure, grouped by separation-ESI mode (C18 positive and negative). Each violin plot represents the distribution of log-transformed ion intensities for a given metabolite at baseline and postfire timepoints. The width of the 'violin' reflects the kernel density estimate, indicating the frequency of values across the distribution. The inner white box shows the interquartile range (IQR), with the horizontal black line inside representing the median. Orange diamonds indicate the mean ion intensity. Solid lines connecting the group means from baseline to postfire illustrate the directionality of change: upward lines suggest upregulation and downward lines indicate downregulation after exposure. Statistical significance was assessed using two-sample Wilcoxon tests, with adjusted q-values (FDR) denoted as follows: q < 0.05 (*), q < 0.01 (**), and q < 0.001 (***). Metabolites without asterisks did not reach statistical significance.


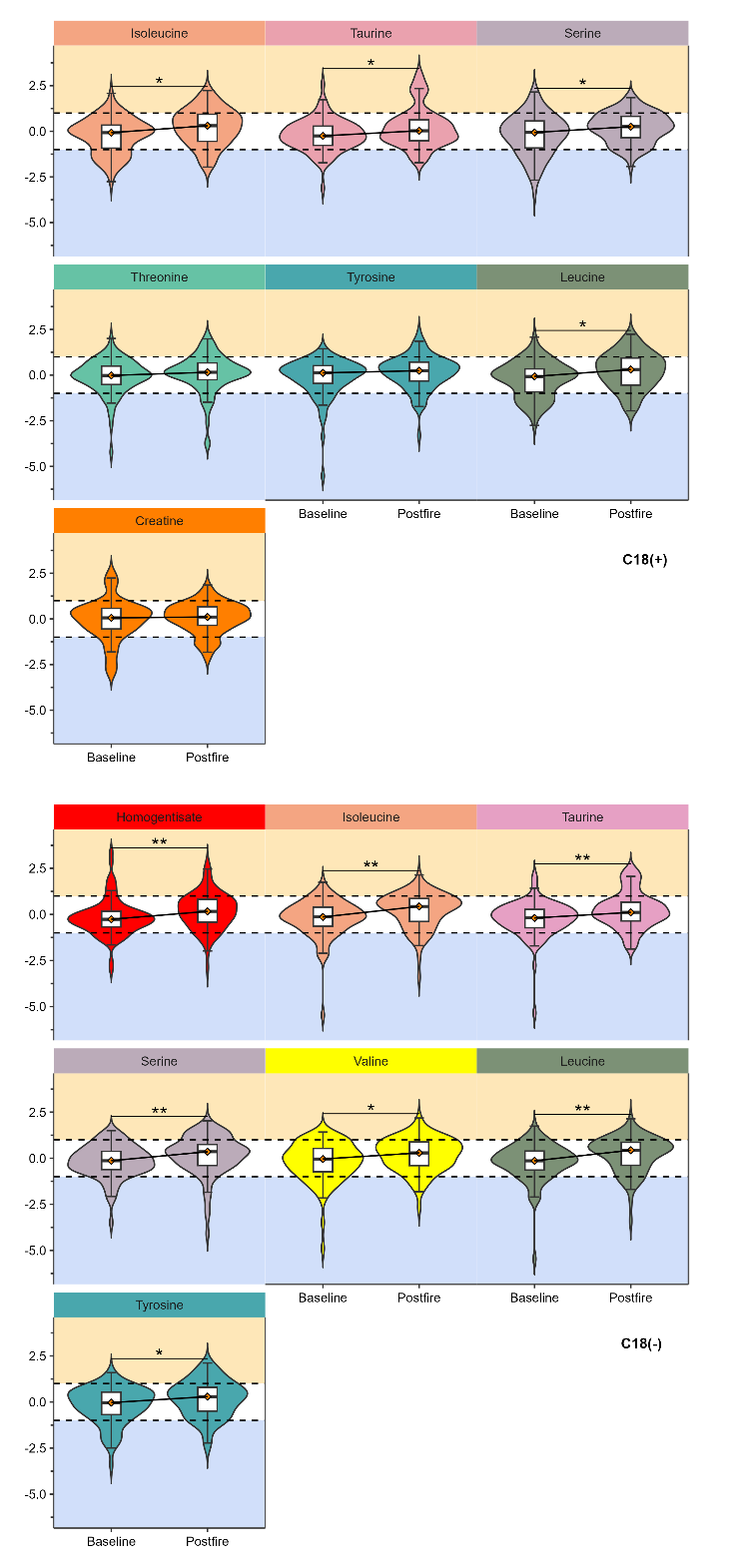


Supplemental Figure 2B. Violin plots of select metabolites involved in enriched pathways following WUI fire exposure, grouped by separation mode (HILIC positive and negative). Each violin plot represents the distribution of log-transformed ion intensities for a given metabolite at baseline and postfire timepoints. The width of the 'violin' reflects the kernel density estimate, indicating the frequency of values across the distribution. The inner white box shows the interquartile range (IQR), with the horizontal black line inside representing the median. Orange diamonds indicate the mean ion intensity. Solid lines connecting the group means from baseline to postfire illustrate the directionality of change: upward lines suggest upregulation and downward lines indicate downregulation after exposure. Statistical significance was assessed using two-sample Wilcoxon tests, with adjusted p-values (FDR) denoted as follows: q < 0.05 (*), q < 0.01 (**), and q < 0.001 (***). Metabolites without asterisks did not reach statistical significance.


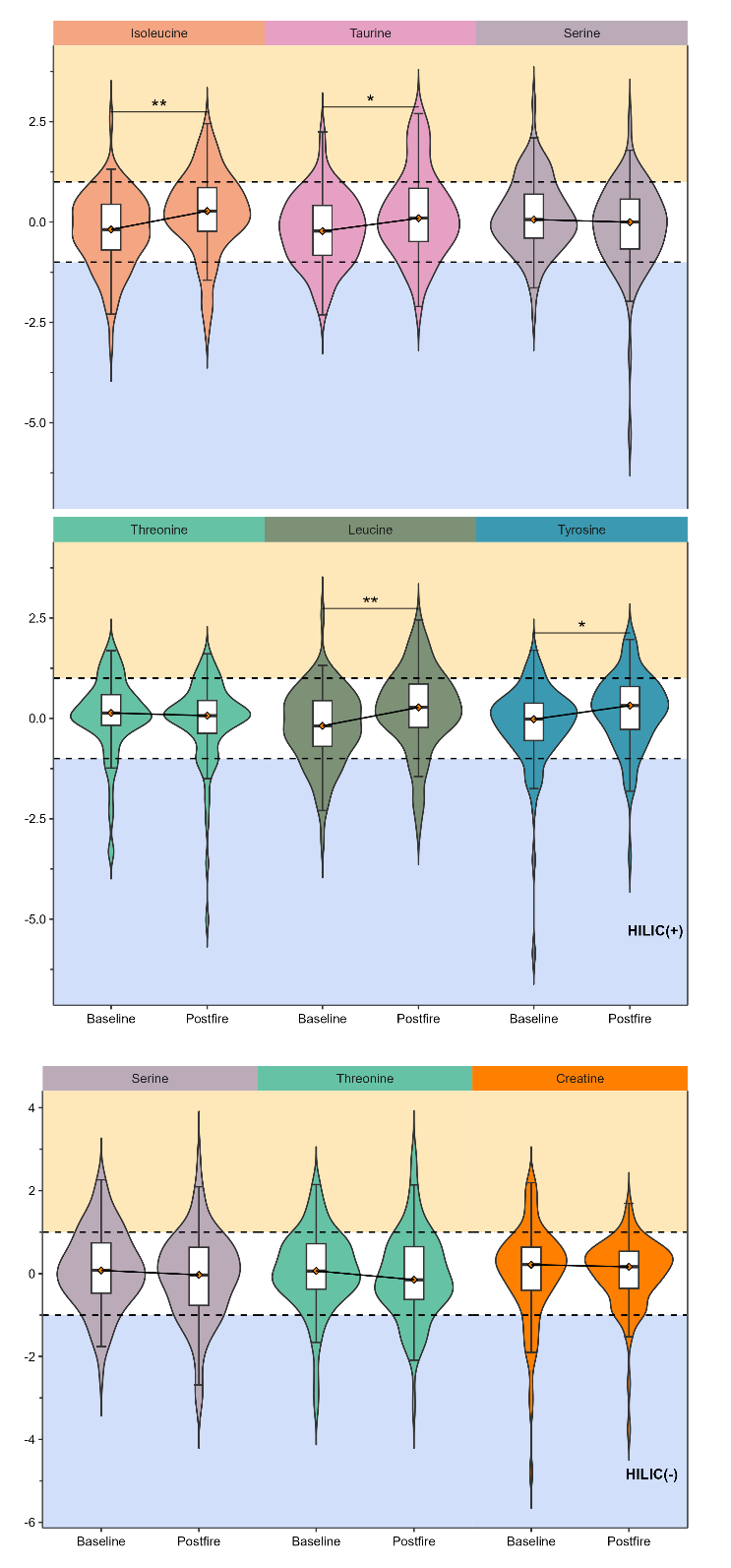


Supplemental Figure 3A. Violin plots comparing excluded metabolites from main model in WUI C18 mode by fire exposures. **Only significantly altered metabolites by fire exposure at p-value 0.05 level were included**. Two sample Wilcoxon tests were performed to derive statistical significance. Statistical significances were indicated as * if the adjusted p-value by controlling family-wise error rate at FDR 0.05 level was less than 0.05. Solid lines between baseline and postfire samples connected two sample median ion intensities to demonstrate the directionality of metabolite regulation after fire exposure. An upward trend showed upregulation while a downward trend indicated downregulation.


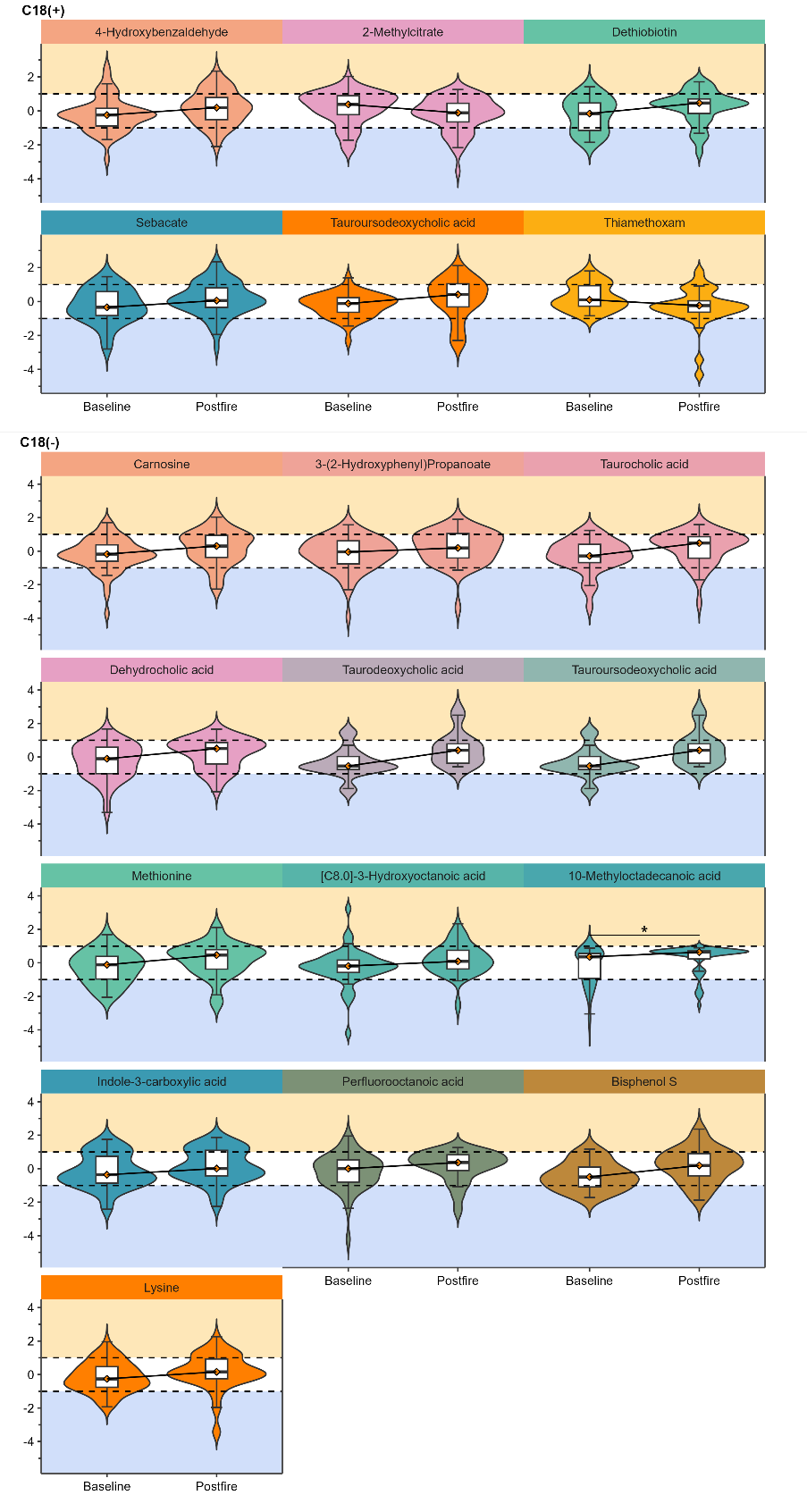


Supplemental Figure 3B. Violin plots comparing excluded metabolites from main model in WUI HILIC mode by fire exposures. Only significantly altered metabolites by fire exposure at p-value 0.05 level were included. Two sample Wilcoxon tests were performed to derive statistical significance. Statistical significances were indicated as * if the adjusted p-value by controlling family-wise error rate at FDR 0.05 level was less than 0.05. Solid lines between baseline and postfire samples connected two sample median ion intensities to demonstrate the directionality of metabolite regulation after fire exposure. An upward trend showed upregulation while a downward trend indicated downregulation.


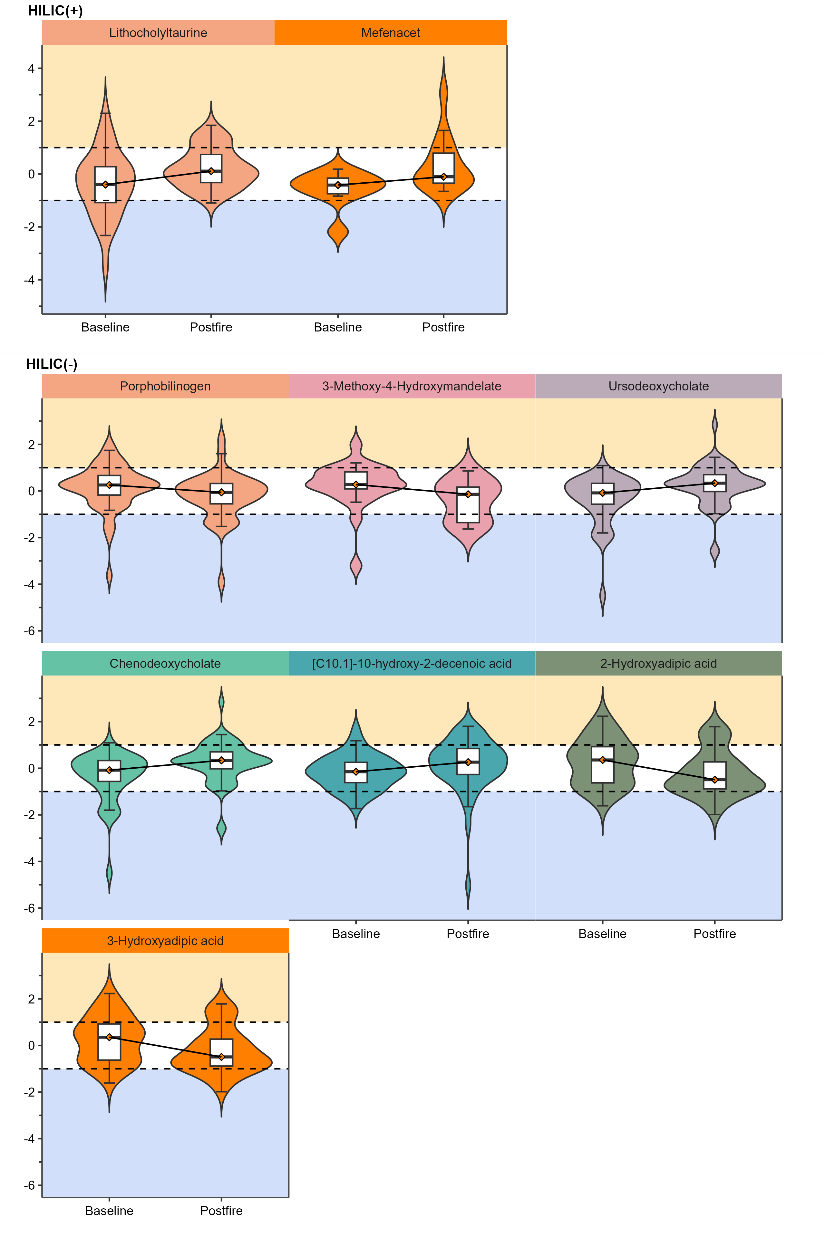


Out of 218 excluded metabolites, a total of 28 metabolites were tested to be significantly altered after WUI fire exposure at the raw p-value of 0.05 level, and only 10-methyloctadecanoic acid reached statistical significance at FDR 0.05 level possibly due to the skewness in the postfire distribution. Overall, the excluded metabolites were not differential by our definition, though tested by a different procedure.
